# Litter Removal Counteracts the Effects of Warming on Soil Bacterial Communities in the Qinghai–Tibet Plateau

**DOI:** 10.3390/microorganisms12112274

**Published:** 2024-11-09

**Authors:** Guanwen Li, Yang Wu, Wenjing Chen, Ziwen Zhao, Yuanze Li, Leilei Qiao, Guobin Liu, Sha Xue

**Affiliations:** 1 College of Forestry, Northwest A&F University, Yangling 712100, China; 2 State Key Laboratory of Soil Erosion and Dryland Farming on the Loess Plateau, Institute of Soil and Water Conservation, Northwest A&F University, Yangling 712100, China; 3 Moutai Institute, Renhuai 564500, China; 4 Institute of Soil and Water Conservation, Chinese Academy of Sciences and Ministry of Water Resources, Yangling 712100, China

**Keywords:** warming, litter removal, microbial community, microbial co-occurrence networks, alpine meadow ecosystem

## Abstract

Climate warming and high-intensity human activities threaten the stability of alpine meadow ecosystems. The stability of the soil microbial community is crucial for maintaining ecological service function. However, the effects of warming and litter removal on microbial interactions, community-building processes, and species coexistence strategies remain unclear. In this study, we used a fiberglass open-top chamber to simulate global change, and moderate grazing in winter was simulated by removing above-ground litter from all plants in the Qinghai–Tibet Plateau, China, to investigate the effects of warming, litter removal, and interactions on soil microbial communities. The treatments included (1) warming treatment (W); (2) litter removal treatment (L); (3) the combined treatment (WL); and (4) control (CK). The results show that compared with the control treatment, warming, litter removal, and the combined treatments increased bacterial Shannon diversity but reduced fungal Shannon diversity, and warming treatment significantly changed the bacterial community composition. Warming, litter removal, and the combined treatments reduced the colinear network connectivity among microorganisms but increased the modularity of the network, and the average path distance and average clustering coefficient were higher than those in the control group. Stochastic processes played a more important role in shaping the microbial community composition, and soil–available phosphorus and soil ammonium contributed more to the βNTI of the bacterial community, while total phosphorus and NAG enzyme in the soil contributed more to the βNTI of the fungal community. Notably, litter removal counteracts the effects of warming on bacterial communities. These results suggest that litter removal may enhance bacterial community stability under warming conditions, providing insights for managing alpine meadow ecosystems in the context of climate change.

## 1. Introduction

Since the industrial revolution, global climate warming caused by the use of fossil fuels and anthropogenic land-use changes has become an increasingly important topic of concern [1]. Extreme climate change (heavy rainfall and extremely high temperatures) caused by human activities has been confirmed, and the resulting ecological problems are often irreversible. The soil microbial community is highly sensitive to temperature as it is a crucial factor affecting the elemental cycle in the soil ecosystem [2]. Temperature increases can directly or indirectly affect the soil microbial communities’ composition [3]. Grazing remains the most common land-use activity in some alpine meadow areas [4]. However, climate warming and grazing will inevitably coincide in alpine meadow ecosystems under future climate scenarios. It is thus necessary to study the effects of warming and litter removal on the soil microbial community structures, interactions, and species coexistence strategies.

Soil microbes play a crucial role in determining the feedback of ecosystems to global warming [5] and have been extensively studied in recent decades [1]. Many simulated warming experiments have shown that warming recombines soil microbial communities [6,7]. This is because warming can directly change the composition of soil microbial communities, such as experimental warming leading to successive changes in soil microbial communities in a tall-grass prairie ecosystem [1]. Furthermore, according to the metabolic theory, warming enhances microbial metabolic activities; however, as soil substrates are gradually consumed, microbial activity is inhibited [3]. Finally, warming changes plant communities and reduces soil moisture, indirectly affecting the composition of soil bacterial and fungal communities [8]. However, the responses of the microbial communities to warming are inconsistent across different ecosystems and timescales. For instance, the bacterial community did not change after short–term warming (4 years) in temperate forest soils [9], but bacterial diversity increased after long-term warming (20 years) [10]. Some studies have found that the fungal diversity of alpine forests and tropical grassland ecosystems does not change with long-term simulated warming, but the community structures do change [6,7]. However, long-term simulated warming was found to significantly reduce microbial biodiversity in grassland ecosystems [8,11], but it did not change substantially in shrub ecosystems [8]. Thus, despite our intensive study on how climate warming affects microbial communities, its impacts on the coexistence of the vegetation–soil–microbiome remain elusive. In addition, global warming affects plant growth, which, in turn, affects the quality of the litter input [8]. Plant litter is an important source of the soil carbon pool, and its quantity and quality have an important impact on dynamic changes in the soil carbon pool [12]. Previous studies have shown that litter removal reduces the availability of substrates for microorganisms and affects biochemical processes in the soil [1], which in turn affects the reorganization and catabolic functions of the soil microbial communities [13,14]. Different microbial communities may respond differently to climate change, even under identical resource conditions [15]. A reduction in vegetation cover can also affect soil pH, temperature, and water content, thereby indirectly affecting microbial metabolic processes [8]. Collectively, both warming and litter removal may significantly affect the soil microbial community structures and interactions, especially in ecosystems susceptible to climate change and human activities [16]. However, the responses of the soil bacterial and fungal community structure and coexistence mechanisms to climate change (warming) in alpine grassland ecosystems under different resource availability states (litter removal) remain elusive [4].

Numerous studies have demonstrated that warming and litter removal can significantly impact the structure and function of specific microbial taxa within grassland ecosystems. For example, Acidobacteria is a common bacterial phylum in alpine soils that is able to decompose complex organic matter and regulate soil pH, which is essential for maintaining nutrient cycling under warming conditions [17]. Warming has been found to enhance the activity of Proteobacteria in alpine grasslands, which is associated with rapid nutrient cycling and adaptation to fluctuating soil moisture levels [18]. In terms of fungal taxa, Ascomycota and Basidiomycota are key players in organic matter decomposition and nutrient transformation. These taxa exhibit distinct responses to warming; Ascomycota tends to thrive under reduced soil moisture and litter removal, likely due to its ability to exploit a wide range of substrates [19]. Meanwhile, Basidiomycota shows resilience to warming but can be sensitive to litter reduction, which limits the availability of complex substrates needed for its growth [20]. In alpine ecosystems, the symbiotic relationships between arbuscular mycorrhizal fungi and plant roots play a critical ecological role, particularly under warming, by facilitating nutrient uptake and providing drought resistance to plants [21]. However, warming and litter removal can alter the abundance and activity of these symbionts, potentially disrupting nutrient dynamics and plant–microbe interactions [22]. Collectively, these shifts in microbial communities not only impact soil biochemical processes but also influence the overall ecosystem resilience to climate change, emphasizing the complex feedback between vegetation, soil, and microbes in response to warming and litter removal in alpine grasslands [23].

The Qinghai–Tibet Plateau is one of the regions most sensitive to climate change and human disturbances, especially grazing [4]. Alpine meadows account for approximately 40% of the grassland area of the Qinghai–Tibet Plateau and contribute significantly to the global soil carbon pool. Almost all alpine meadows are used for grazing. Assessing the response of soil bacterial and fungal communities and critical taxa to warming and litter removal in the alpine meadow ecosystem of the Qinghai–Tibet Plateau has important implications for our understanding of the feedback of alpine meadow ecosystems on global climate change and human activities. We have thus conducted in situ warming and litter removal experiments for seven consecutive years in the alpine meadow ecosystem of the Qinghai–Tibet Plateau (Haibei Experimental Station, Haixi Prefecture, Qinghai Province, China). It was hypothesized that (1) warming and litter removal would change the soil bacterial and fungal community structures and community building processes in the ecosystem, and bacterial and fungal communities would respond differently to warming and litter removal, which is related to resource availability, and (2) that warming and litter removal would also reshape the interaction network among microorganisms, independent of microbial diversity, and the fungal network would have a higher resistance to warming and litter removal.

## 2. Materials and Methods

### 2.1. Experimental Plot

The field experiment was conducted at the Haibei Research Station in Qinghai Province, China (37°36′ north, 101°19′ east), which is an ecological observation station integrating long-term observations, research, and testing. The experimental station’s average annual temperature and precipitation were −1.7 °C and 561 mm, respectively [24]. The primary soil type was Mollic–Cryic Cambisol, the vegetation was mainly alpine Kobresia meadows, and the dominant species included *Stipa heterophylla*, *S. superba*, *Leontopodium nanum*, *Potentilla nivea*, and *Gentiana straminea*. The vegetation coverage was approximately 80% when there was vigorous growth (August). The experiments commenced in 2011, and a block and split-zone design was adopted that used four treatments: warming (W), litter removal (L), combined (WL), and control (CK), with five replicate plots per treatment. Each small plot was 1.77 m^2^, with a 2 m interval between each plot, and they were protected from animals trampling and gnawing. Open-top chambers (OTCs) were used to simulate climate warming. The daytime and average temperatures increase by 1.58–5.24 °C and 1.22–4.28 °C, respectively. Dead leaves were removed (litter removal) from the ground by hand-picking in April or early May of each year to simulate proper grazing during the winter.

### 2.2. Soil Collection and Analysis

Soil samples were collected and tested at the field experiment station in August 2018. After removing the surface materials, 10 soil cores (5 cm in diameter and 5 cm in depth) were randomly collected from each plot and thoroughly mixed after removing excess roots and stones. A total of 20 soil samples were collected from 4 treatments. The measurement methods of the soil total organic carbon (SOC), total nitrogen (TN), total phosphorus (TP), nitrate N (${NO}_{3}^{-}$–N), soil ammonium (${NO}_{4}^{+}$–N), soil pH, and soil available phosphorus (AP) are as previously described in [24]. The activity of the soil ecological enzymes related to the C, N, and P cycles was measured using a fluorescent microplate method, including β-glucosidase (BG), β–xylosidases, cellulose (CELL), leucine–α–aminopeptidase (LAP), N–acetyl–β–d–glucosaminidase (NAG), alkaline phosphatase (AP), and alanine aminopeptidase (ALT). The richness and diversity of plant communities were recorded and measured by placing a 10 × 10 grid sample frame (0.25 m^2^) above the vegetation crown layer, identifying all species in the quadrat in the field, and taking photos for archiving.

### 2.3. Microbial Data and Analysis

Soil microbial DNA was extracted from 0.25 g of frozen soil using a Power Soil DNA Separation Kit (MO BIO Laboratories, Beijing, China). A NanoDrop 2000 spectrophotometer (Thermo Scientific, Waltham, MA, USA) was used to estimate the DNA concentrations, and the DNA sequence quality was determined with 1% agarose gel electrophoresis [25]. The purified soil DNA was stored in a −80 °C freezer. The bacterial 16S rRNA gene (V3-V4 region) was amplified using the forward primer 338F (5′-ACTCCTACGGGAGGCAGCA-3′) and reverse primer 806R (5′-GGACTACHVGGGTWTCTAAT-3′). The ITS1 region of the fungi was amplified using the forward primer ITS1F (5′-CTTGGTCATTTAGAGGAAGTAA-3′) and reverse primer ITS2-2043R (5′-GCTGCGTTCTTCATCGATGC-3′). High-throughput sequencing was performed on a total of 20 samples using the Illumina NovaSeq platform (Beijing, China), with sequence quality filtering using QIIME 2 platform [26]. The arrangements were clustered using UPARSE software (http://drive5.com/uparse/, accessed on 20 June 2022), and the amplicon sequence variants were replaced with 97% of the OTUs. The bacteria and fungi were determined using RDP classifiers (http://www.arb-silva.de, accessed on 20 June 2022) from the Silva Release 138.1 and Unite 9.0 database (https://unite.ut.ee/, accessed on 20 June 2022).

### 2.4. Network Construction

We constructed microbial co-occurrence networks using the Molecular Ecological Network Analysis (MENA) pipeline (http://ieg4.rccc.ou.edu/mena, accessed on 25 July 2022) to examine interactions among microbial taxa under different treatments. Rare OTUs with a relative abundance of <0.01% were excluded to minimize noise and highlight significant interactions. Only OTUs with a Spearman correlation coefficient (ρ) > 0.7 and a false discovery rate-corrected *p*–value < 0.01 were retained for analysis, ensuring reliable network structure. Based on Stochastic Matrix Theory (RMT) [27], we performed Spielman correlation analysis using log-transformed OTUs to construct bacterial and fungal networks under the four treatments, focusing on bacteria-fungus interactions under warming and litter removal. The similarity threshold was set at 0.98 to ensure robustness [3]. In these networks, nodes represent OTUs, while edges indicate significant correlations between OTUs, reflecting potential microbial interactions. Key metrics calculated included average path distance (GD), average degree (avgK), average clustering coefficient (avgCC), transitivity, and modularity. These metrics help to infer network complexity and connectivity: a higher avgK, avgCC, and transitivity suggest stronger connectivity among microbial taxa, indicating potential mutualism or cooperation [28]. Lower GD and modularity reflect more integrated networks, which could suggest stronger functional resilience under environmental perturbations. Then, the bacterial and fungal network data calculated based on MENA were merged and visualized using Gephi 0.9.2. We also generated 100 random networks to validate the empirical network’s significance, ensuring the observed patterns were not random artifacts. The rationale for using network analysis is that it provides insights into how warming and litter removal may reshape microbial community interactions, which is critical for understanding ecosystem responses to global change. It helps identify whether these treatments enhance positive or negative microbial associations, providing valuable information about ecosystem stability and function [29]. 

### 2.5. Community Assembly Processes

To explore the internal dynamics of microbial community construction, we employed the null model–based iCAMP approach, which quantifies the relative importance of different community assembly processes, such as variable selection, heterogeneous selection, dispersal limitation, and drift [30]. The β Nearest Taxon Index (βNTI) was used to determine niche preferences [31,32,33], while the modified Raup–Crick index (RC) assessed β diversity. Specifically, βNTI < −2 and βNTI > 2 indicate homogeneous and heterogeneous selection, respectively. For |βNTI| ≤ 2, the RC index further categorizes the processes into homogeneous diffusion (RC < −0.95), diffusion limitation (RC > 0.95), and drift (|RC| ≤ 0.95). This approach allows us to differentiate between deterministic and stochastic factors driving microbial community changes. For instance, a higher influence of homogeneous selection would suggest that specific environmental conditions, such as nutrient availability, shape the community, while higher dispersal limitation or drift indicates that random processes are more significant. This is crucial for understanding how warming and litter removal impact microbial community composition and stability, which is essential for managing ecosystem functions [34]. Analyses of soil microbial construction processes were performed using vegan (v2.6.4) [35] and iCAMP (v1.3.4) packages in R3.6.3 [30].

### 2.6. Statistical Analysis

We performed α diversity and β diversity analyses using sequencing data to assess microbial diversity under different treatments. We used the Bray–Curtis index to perform principal coordinates constraint analysis (CAP) and permutational multivariate analysis of variance (PERMANOVA) to visualize and quantify differences in β diversity [36]. OTU sequences were normalized using the “Pruning Mean of M” (TMM) method in the Bioconductor package edgeR (v4.0) [37], enabling accurate comparison across treatments. Random forest analysis was used to identify key variables influencing microbial abundance, with Gini index reduction used to assess variable importance. The Kruskal–Wallis test was conducted on the top 10 variables, identifying statistically significant differences. We also employed the Mantel test to examine correlations between microbial diversity, soil nutrients, and vegetation diversity, providing insights into the potential drivers of microbial community changes. PLS–PM was used to identify the direct and indirect effects of experimental treatments on soil physicochemical properties, vegetation diversity, and microbial Shannon diversity, as well as microbial co-occurrence networks, using the “plspm” (v0.4.1) package in R4.0.3 [38]. This holistic statistical approach helps disentangle complex interactions, showing how warming and litter removal directly and indirectly shape microbial dynamics. The above statistical analyses were performed using R3.6.3 and R4.0.3 (https://www.r-project.org/). 

## 3. Results

### 3.1. Soil Microbial Diversity and Community Structure 

**Figure 1:**
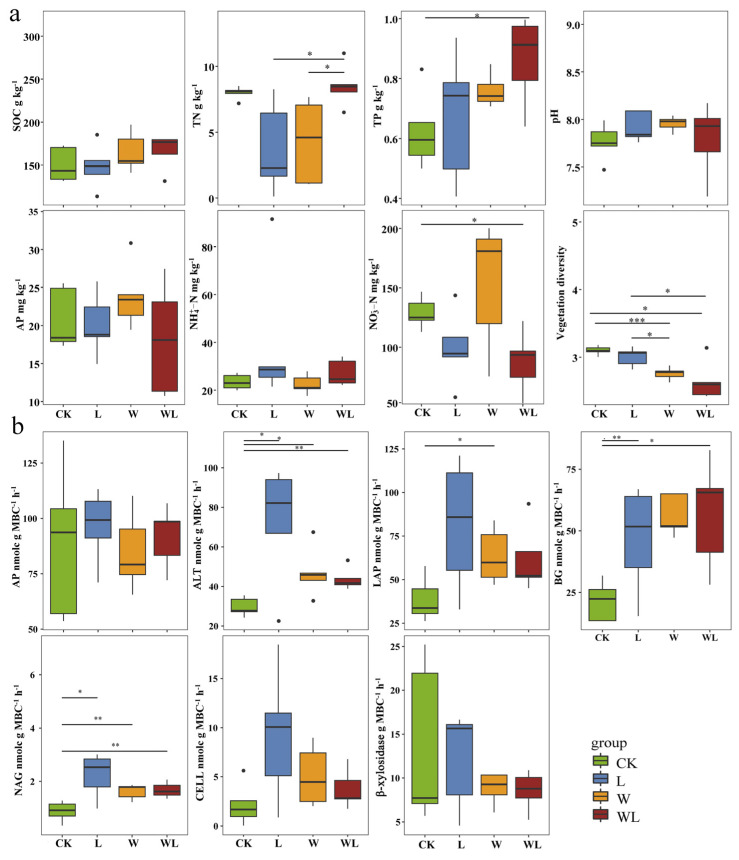
Effects of warming and litter removal on soil properties and enzyme activities. Kruskal–Wallis rank-sum test of soil properties among treatments. SOC, soil total organic carbon; TN, soil total nitrogen; TP, soil total phosphorus; AP, available phosphorus; soil ammonium (${NO}_{4}^{+}$–N); and nitrate (${NO}_{3}^{-}$–N) (**a**). Kruskal–Wallis rank-sum test of enzyme activities among treatments. AP, acid phosphatase; ALT, alanine aminopeptidase; LAP, leucine aminopeptidase; BG, β-1,4-glucosidase; NAG, N-acetyl-β-D-glucosaminidase; CELL, cellulase (**b**). Significant effects are indicated in bold (* *p* < 0.05, ** *p* < 0.01, *** *p* < 0.001). WL, warming + litter removal; W, warming; L, litter removal; CK, control.

**Figure 2:**
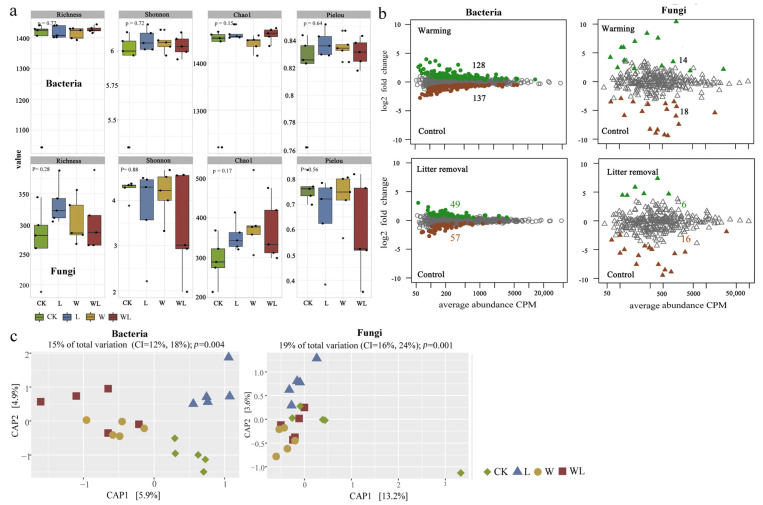
Effects of warming and litter removal on soil microbes. Alpha diversity of soil bacteria and fungi among different treatments (**a**). Microbiota specific to soil bacteria and fungi under warming and litter removal conditions compared with the control (**b**). The *X*-axis represents the mean OTU abundance (per million, CPM), and the *Y*-axis represents the log2 fold change (warming relative to the control). Warming- and control-specific OTUs are in green and brown, respectively, while OTUs with no differential abundance are in grey (likelihood ratio test, *p* < 0.05, FDR corrected). Effects of warming and litter removal on the composition of soil bacterial and fungal communities (**c**). WL, warming + litter removal; W, warming; L, litter removal; CK, control.

Litter removal (L) and the combined treatment (WL) significantly reduced vegetation diversity and the soil ${NO}_{3}^{-}$–N content but increased the soil TP content (Figure 1a). The L and WL treatments also considerably increased the enzyme activities of ALT, BG, and NAG related to carbon and nitrogen cycles (Figure 1b). In addition, compared with the CK, the W, L, and WL treatments increased bacterial alpha diversity (Shannon, Chao1, Pielou) and decreased fungal Shannon diversity, but neither was significant (Figure 2a). Warming increased the relative abundance of bacteria Acidobacteria and Actinobacteria, while W, L, and WL treatments decreased the relative abundance of fungi Mortierellomycota and Basidiomycota but increased the relative abundance of Ascomycota (Figure S1). The W, L, and WL treatments also showed different microbial habitats (Figure 2b). The W and WL treatments significantly increased the number of specific OTUs in Proteobacteria, such as *uncultured_bacterium_g_Sphingopyxis*, *uncultured_bacterium_g_Zymomonas*, and *uncultured_bacterium_g_Pseudomonas*, but decreased the number of specific OTUs in Planctomycetes and Nitrospirae, such as *uncultured_bacterium_c_OM190*, *uncultured_bacterium_f_Planctomycetaceae*, *uncultured_bacterium_g_SM1A02*, and *uncultured_bacterium_f_0319-6A21*. In addition, fungal-specific OTUs, such as *Mortierella_elongata* and *Mucor_hiemalis*, were increased in the W and WL treatments, as well as specific OTUs in the CK treatment, such as *Cordyceps_bassiana*, *Hygrocybe_conica*, and *Vishniacozyma_victoriae*. The Constrained Analysis of Principal Coordinates (CAP) showed that the W and WL treatments formed different clusters from the CK and L treatments in the bacterial community, while W and WL could not be distinguished. In the fungal community, the W and L treatments clustered more clearly, and the WL and CK treatments could not be distinguished (Figure 2c). PERMANOVA analysis also showed that the warming (W, WL) significantly changed the composition of the soil bacterial community, and litter removal (L, WL) had little effect on the composition of the microbial community (Table 1). BETADISP was used to test the dispersion differences in soil microbial communities, and there were also significant differences in soil bacterial communities between warming (W, WL) (Table 1). Pairwise treatment tests showed that warming alone significantly changed the composition of bacterial communities (*p* < 0.05), while there was no significant difference in bacterial communities between the combined treatment and the control, and litter removal offset the effect of warming on the composition of soil bacterial communities.

### 3.2. Interaction Patterns of Soil Microorganisms and Microbial Community Assembly Processes

**Figure 3:**
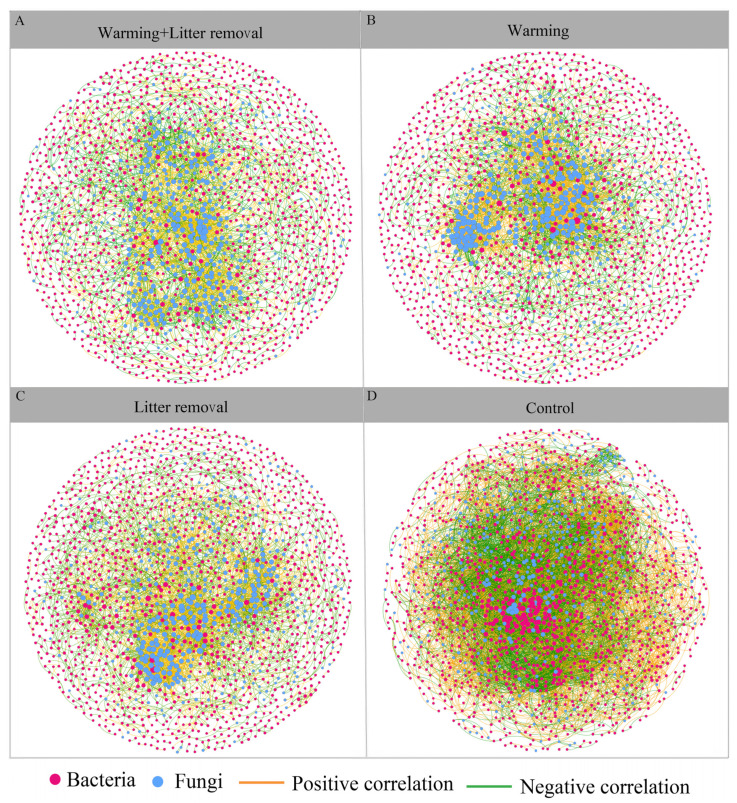
Co-occurrence network interactions of the soil bacterial and fungal communities at the OTU level. The co-occurrence network was visualized based on Gephi 0.9.2 software.

The molecular ecological networks revealed that under CK, the microbial community had a higher average degree (avgK), average clustering coefficient (avgCC), and a lower average path distance (GD), as shown in Table 2. This suggests that microorganisms in the control group were more densely connected and that the network exhibited greater scale, complexity, and modularity. Warming alone and litter removal alone treatments reduced the number of positive and negative correlations between bacteria and fungi. Interestingly, compared with the warming alone, the combined treatment led to an increase in positive correlations between fungi, a decrease in negative correlations, and strengthened mutualistic interactions among fungal communities (Figure 3 and Table 2). Furthermore, the modularity values across all treatments ranged from 0.563 to 0.724, all of which were significantly higher than the corresponding randomized networks (Table 2). This indicates that all the constructed molecular ecological networks (MENs) maintained a modular structure. The connectivity between nodes within and between modules (Supplementary Figure S3), quantified by the intramodule connectivity (Zi) and intermodule connectivity (Pi), showed that most nodes (~96.5%) in the warming and litter removal treatments (W, L, WL) were classified as peripherals. In the microbial co-occurrence network of the W treatment, 18 nodes were “module hubs”, and 14 modules were “connectors”. The microbial co-occurrence network of the L treatment had 30 nodes as “module hubs” and 42 nodes as “connectors”. The microbial co-occurrence network of the combined treatment had 14 nodes as “module hubs” and 28 nodes as “connectors” (Supplementary Table S1). *Proteobacteria* and *Acidobacteria* had module connection roles in the bacterial co-occurrence network, while *Ascomycota* had module connection roles in the fungal co-occurrence network.

**Figure 4:**
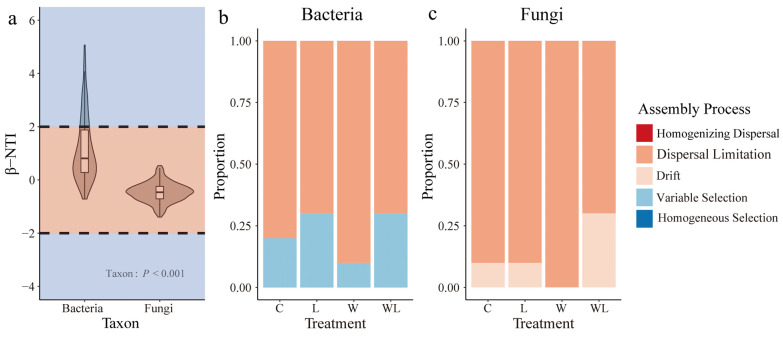
The assembly process for soil bacterial and fungal communities in “Litter removal” and “Warming” treated. Distribution of βNTI in bacteria and fungi (**a**) and proportion of assembly processes in bacterial (**b**) and fungal (**c**) communities. In (**a**), expectations are less than zero (i.e., βNTI < −2), showing homogeneous choice; expectations being more than expected zero (i.e., βNTI > 2) indicates that the variable selection, and phylogenetic turnover does not deviate from the desired zero (|βNTI| < 2) indicates neutral process. *p* values from Kruskal–Wallis test (**a**). WL, warming + litter removal; W, warming; L, litter removal; CK, control.

Null model analysis (iCAMP) revealed that bacterial and fungal communities were predominantly composed of stochastic processes, with variable selections having more significant importance in the bacterial community structure, while random drift had more significance in the fungal community structure (Figure 4). Specifically, the bacterial community was mainly composed of dispersal limitation and variable selection. In the L and WL treatment, the bacterial community showed higher variable selection, while the fungal community was mainly composed of dispersal limitation and drift, and the WL treatment increased the drift process of the fungal community (Figure 3B,C). Variation partitioning revealed that soil accounted for a large proportion of the variation in the bacterial and fungal community βNTI (Table 3). The importance of variable selection in constructing bacterial communities was related to specific soil properties (such as AP and ${NO}_{3}^{-}$–N availability), and fungal community drift was related to vegetation and soil properties (TP availability).

**Figure 5:**
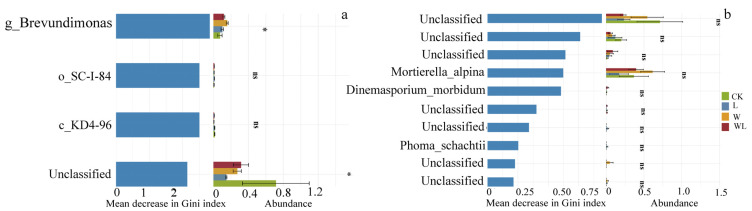
(**a**,**b**) Combinatorial plot of test for differences in the importance of variables and species abundance between random forest groups. Kruskal–Wallis test was performed on the top 10 important variables of random forest analysis, and significant markers were extracted (*p* > 0.05, “ns”, *p* < 0.05, “*”).

The key OTUs (Figure 5) selected by the bacteria were *Brevundimonas* (Proteobacteria), *SC-I-84* (Proteobacteria), *KD4-96* (Chloroflexi), and *Unclassified* (Acidobacteria), and the *KD4-96* and *Brevundimonas* were significantly different. The ten key OTUs selected by the fungi were not significant. Warming decreased the abundance of fungal *Unclassified* (Ascomycota) but increased the abundance of *Mortierella_alpina* (Mortierellaalpina).

### 3.3. Driving Factors of Microbial Community Change

**Figure 6:**
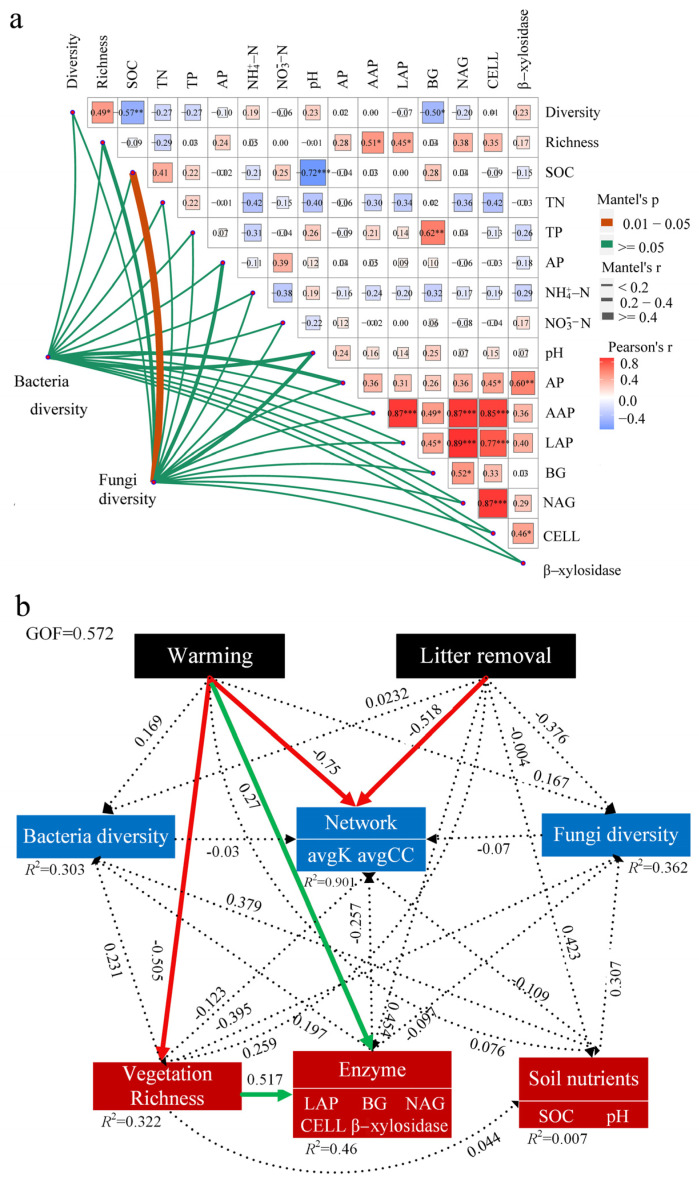
Environmental drivers of microbial diversity. Correlation between environmental variables and microbial diversity (**a**) (* *p* < 0.05, ** *p* < 0.01, *** *p* < 0.001). PLSPM shows the relationship between vegetation abundance, soil properties, and soil enzyme activities with microbial diversity and a collinear network (**b**). Green and red arrows indicate positive and negative relationships, respectively. Solid or dashed lines indicate a significant (*p* < 0.05) or insignificant relationship.

The Mantel test showed that bacterial and fungal Shannon diversity indices were slightly correlated with vegetation abundance, soil organic carbon, and pH (Figure 6a). However, only the fungal Shannon diversity index was significantly positively correlated with SOC. To further clarify the direct and indirect effects of warming and litter removal on microbial diversity and co–occurrence networks, a PLSPM analysis was performed (Figure 6b). Warming and litter removal significantly reduced the complexity of the bacterial (normalized path coefficient, b = −0.75, *p* < 0.05) and fungal (b = −0.518, *p* < 0.05) network. Warming could significantly and directly affect the activities of extracellular soil enzymes (LAP, BG, NAG, CELL, and β–xylosidases) (*p* < 0.05) and can also indirectly affect the activities of extracellular soil enzymes (*p* < 0.05) by significantly reducing vegetation diversity. However, warming, litter removal, soil physical and chemical properties, and vegetation abundance did not significantly or indirectly affect the bacterial or fungal Shannon diversity indices. The bacterial and fungal diversity Shannon indices had less impact on the microbial co–occurrence networks, further verifying that the symbiotic networks of the bacteria and fungi are independent of their abundance and connectivity data. Furthermore, PLSPM analysis revealed that warming and litter removal played dominant roles in the formation of microbial co-occurrence networks (R^2^ = 0.9).

## 4. Discussion

### 4.1. Responses of the Soil Microbial Community to Warming and Litter Removal

Previous studies have shown that warming and changes in the soil and vegetation caused by warming can alter the composition and diversity of microbial communities [7,39,40]. The increase in bacterial alpha diversity, notably Acidobacteria and Actinobacteria under W and WL treatments, reflects their roles in nutrient cycling and organic matter decomposition. The results are consistent with previous experiments conducted at the same site [4,38]. Warming alone also significantly altered bacterial communities, while fungal communities did not differ between treatments (Table 1). Acidobacteria, abundant in various soil ecosystems, contribute significantly to biogeochemical processes like carbon turnover, exopolysaccharide production, and plant growth promotion. These traits make them potential indicators of soil health under climate change [17,41]. Similarly, Actinobacteria play a key role in decomposing recalcitrant organic matter, promoting nutrient availability, and enhancing soil resilience, aligning with their known roles in agroecosystems [41]. Specific OTUs, such as *Sphingopyxis* and *Zymomonas* under W and WL treatments, suggest enhanced degradation of aromatic compounds [42]. These taxa’s adaptability to changing environments underscores their functional importance in disturbed soils. Fungal communities showed a strong tolerance to warming and litter removal [43,44,45]. This suggests that fungal communities may be less sensitive to temperature-induced changes in resource availability, while bacterial communities are more responsive to warming-induced changes in nutrient cycling, possibly due to increased soil microbial activity. Huang et al. [15] also showed that bacterial communities are more sensitive to soil pH and resource availability and contribute twice as much to changes in bacterial community structure as fungal communities. The rise of Ascomycota under W, L, and WL treatments emphasizes their dominance as decomposers of cellulose and lignin-rich substrates, supporting organic matter turnover [46]. The decline in Mortierellomycota and Basidiomycota may suggest reduced nutrient exchange and mycorrhizal associations, consistent with findings in other disturbed ecosystems [47]. In addition, interactions between bacteria and fungi are more conducive to microbial community stability. For example, hyphal fungi can promote bacterial activity by providing energy under resource stress, and this interaction helps maintain the stability of ecosystems in stressed habitats [48]. Overall, these shifts indicate that Acidobacteria, Proteobacteria, and Ascomycota can serve as microbial indicators of ecosystem health and bioremediation potential, supporting their functional roles in maintaining soil resilience under warming scenarios [41].

### 4.2. Effects of Warming and Litter Removal on the Microbial Co-Occurrence Network

Litter removal reduced vegetation cover and diversity, while warming increased the degradation of organic soil compounds. These changes in environmental conditions impacted microbial interactions, as evidenced by co-occurrence network analysis [4]. In this study, warming and litter removal had greater effects on the co–occurrence network of fungal communities than on bacterial communities. Fungal networks under warming, litter removal, and the combined treatments had more links than the control and showed greater connectivity and modularity (Table 2 and Figure 3), indicating stronger interspecific relationships that may be driven by nutrient competition [49]. This pattern implies that fungal communities may establish more stable interaction networks under altered resource conditions, maintaining ecosystem function despite environmental changes. In contrast, bacterial networks showed reduced connectivity (Table 2) under warming, litter removal, and their combination. This suggests that bacterial interactions may be more sensitive to these treatments, which could weaken microbial stability. The combined treatment did not significantly alter negative correlations among nodes, although transitivity and modularity were slightly higher. The combined treatment does not drastically change the network’s core structure but may enhance its stability through increased modularity [3]. In contrast, the warming and litter removal treatment exhibited a slightly higher module value, with more nodes playing key roles and more vital interactions between uses from different modules in the co-occurrence network [49].

The modularity and connectivity of microbial networks provide an important ecological basis for the restoration of alpine meadow ecosystems. When environmental conditions change, these highly interconnected microbial communities can respond quickly, adjust their ecological functions, and promote the restoration of ecological balance [50,51,52]. Through Zi–Pi analysis, we identified Proteobacteria and Acidobacteria as key nodes for module connections in bacterial networks, which not only enhanced the complexity and stability of the network but also improved the adaptability of the entire microbial community under environmental changes. For example, previous studies have shown that Proteobacteria exhibits strong ecological adaptability and metabolic diversity under resource competition and environmental stress, enabling it to establish close ecological interactions with other microbial groups in extreme environments such as alpine meadows [53]. Similarly, Ascomycota plays an important role in ecological functions as a module connection point in the fungal network. The symbiotic network formed by Ascomycota and plant roots not only improves the ability of plants to adapt to harsh environments but also effectively promotes plant growth [54]. Ascomycota has made important contributions to global microbial ecology, especially in cold soils, by promoting decomposition and nutrient mineralization [55]. The key position of these highly connected groups in microbial networks enables them to act as ecological regulators in alpine meadow systems, helping to mitigate the negative effects of climate change and vegetation change.

### 4.3. Microbial Community Construction Process

The microbial community construction processes could advance our understanding of the relationship between microbial communities and ecological strategies that coexist with environmental changes. In this study, bacterial and fungal communities were mainly composed of stochastic processes. Dispersal limitations seem to influence the differences in microbiota assembly [56,57,58,59]. Partial dbRDA–based variation partitioning analysis indicated that a large part of the variation in the bacterial and fungal communities assembly processes could not be explained by the measured environmental variables (Table 3), suggesting that stochastic or unmeasured environmental variables may play a more critical role than deterministic processes in shaping microbial communities under long-term warming [1,34]. Interestingly, warming alone increases the relative importance of dispersal limitation in shaping bacterial community assembly, and the high abundance and rapid growth potential of bacteria contribute to the rapid adaptations observed with climate change and the maximum retention of the original community compositions [15]. Conversely, litter removal alone acted more as a deterministic filter, promoting specific bacterial taxa like *KD4-96*, which are known for their rapid growth in response to resource changes. The combined treatment further increased the role of deterministic selection in bacterial communities, highlighting the complexity of assembly processes under simultaneous warming and litter removal. These findings help us to better understand the response and maintenance mechanisms of soil bacterial communities to climate warming and grazing activities.

Additionally, in our study, bacterial communities had fewer dispersal limitations, more substantial environmental selection, and more positive dominance networks than fungi (Table 2). However, soil bacterial communities are more sensitive to changes in resource availability. Among the environmental factors measured, especially AP, ${NO}_{3}^{-}$–N [11,15] was the leading environmental factor in the process of bacterial community construction (*p* < 0.005). However, some unmeasurable ecological factors, such as soil temperature and moisture, have diffusion-limiting effects on soil fungi [1,60]. However, owing to the complex interactions among these assembly processes, further research will be required to characterize how these processes shape community assembly.

## 5. Conclusions

The effects of warming alone on the bacterial community in the alpine meadow ecosystem could be counteracted by litter removal (simulating proper grazing). Furthermore, the fungal community showed a more substantial tolerance to warming and litter removal, which may be related to a stable soil nutrient supply. Simultaneously, the activity of extracellular enzymes in relation to the carbon, nitrogen, and phosphorus cycles and the stability of the co-occurrence network of soil microorganisms was also increased by removing litter in a climate warming scenario. These results highlight that the antagonism between warming and litter removal is beneficial to the stability of soil microbial communities and that proper grazing or litter removal is conducive to the stability of the alpine meadow ecosystem when also considering climate warming.

## Figures and Tables

**Table 1:** PERMANOVA tests showing the effects of warming and litter removal on bacterial and fungal communities. Pairwise comparison between different treatments (*p* < 0.05). Results of the BETADISP detection of diversity and dispersion differences between bacterial and fungal communities. Significant effects are indicated in bold (* *p* < 0.05, ** *p* < 0.01). WL, Warming + Litter removal; W, Warming; L, Litter removal; CK, Control.

	Bacteria		Fungi	
	Pseudo-F	R^2^	Pseudo-F	R^2^
(W + WL) vs. (L + CK)	1.651 **	0.083	1.082	0.057
(WL + L) vs. (W + CK)	0.869	0.044	0.871	0.046
All sample type	1.282	0.065	0.796	0.042
Pairwise Comparisons				
	WL vs. W (*p* > 0.05)		WL vs. W (*p* > 0.05)	
	WL vs. L (*p* > 0.05)		WL vs. L (*p* > 0.05)	
	WL vs. CK (*p* > 0.05)		WL vs. CK (*p* > 0.05)	
	W vs. L (*p* > 0.05)		W vs. L (*p* > 0.05)	
	W vs. CK (*p* < 0.05)		W vs. CK (*p* > 0.05)	
	L vs. CK (*p* > 0.05)		L vs. CK (*p* > 0.05)	
Multivariate homogeneity of group dispersions				
(W + WL) vs. (L + CK)	2.173 *		0.091	

**Table 2:** Major properties and associated random networks of the microbial networks with the warming and litter removal treatments. B-B, links between bacteria and bacterial nodes; F-F, links between fungi and fungi nodes; B-F, links between bacterial and fungi nodes; F-B, links between fungi and bacterial nodes. WL, Warming + Litter removal; W, Warming; L, Litter removal; CK, Control.

	WL		W		L		CK	
Pearson threshold	0.99		0.99		0.99		0.99	
Nodes	1693		1640		1727		1673	
Links	4423		4523		4882		11005	
Average degree (avgK)	5.225		5.5158		5.6537		13.156	
Randomized network structure	Empirical	100 Random	Empirical	100 Random	Empirical	100 Random	Empirical	100 Random
Average path distance (GD)	7.097	4.259 ± 0.017	6.559	4.088 ± 0.018	6.491	4.117 ± 0.015	4.519	3.183 ± 0.008
Average clustering coefficient (avgCC)	0.297	0.009 ± 0.002	0.304	0.012 ± 0.002	0.322	0.010 ± 0.001	0.351	0.020 ± 0.001
Transitivity (Trans)	0.19	0.012 ± 0.001	0.187	0.016 ± 0.001	0.178	0.012 ± 0.001	0.378	0.026 ± 0.001
Modularity (fast_greedy)	0.71	0.424 ± 0.003	0.691	0.405 ± 0.002	0.724	0.401 ± 0.003	0.563	0.227 ± 0.003
R square of power-law	0.824		0.865		0.837		0.778	
B-B positive correlation	792		800		1229		3491	
B-B negative correlation	744		604		525		2899	
F-F positive correlation	460		339		405		357	
F-F negative correlation	1257		1336		1173		727	
B-F positive correlation	471		578		642		1924	
B-F negative correlation	506		616		908		1607	
F-B positive correlation	97		76		-		-	
F-B negative correlation	96		174		-		-	

**Table 3:** Partitions of βNTI variations according to the soil variables and vegetation communities. The significance of each variable in a community assembly partition was tested based on distance-based redundancy analysis (anova.cca function, vegan package).

Taxon	Partition	Adj.R^2^	*p*-Value	Significant Variables
Bacteria	Soil	0.488	0.005	$\mathrm{AP},\;{NO}_{4}^{+}$–N
	Vegetation	−0.174	0.77	
	Soil + Vegetation	0.273	0.07	
	Soil|Vegetation	0.447	0.018	AP
	Vegetation|Soil	−0.214	0.941	
	Soil ∩ Vegetation	−0.041		
	Residuals	0.727		
Fungi	Soil	0.177	0.011	TP, NAG
	Vegetation	−0.009	0.554	
	Soil + Vegetation	0.198	0.018	
	Soil|Vegetation	0.207	0.008	TP, NAG
	Vegetation|Soil	0.021	0.315	
	Soil ∩ Vegetation	−0.03		
	Residuals	0.802		

## Data Availability

The raw data supporting the conclusions of this article will be made available by the authors on request.

## Supplementary Materials

The following supporting information can be downloaded at https://www.mdpi.com/article/10.3390/microorganisms12112274/s1, Figure S1: Changes in relative abundance of microbial communities under different treatments. Figure S2: Specific microbial communities in soil under different treatments. Figure S3: Zi-Pi plots show the distributions of OTUs based on their topological roles in fungi and bacteria networks. Table S1: Module hubs and connectors nodes filtered by Zi-Pi.
