# Supplementary material for: Litter Removal Counteracts the Effects of Warming on Soil Bacterial Communities in the Qinghai–Tibet Plateau
Source: Microorganisms. 2024 Nov 9;12(11):2274. doi: 10.3390/microorganisms12112274 (PMC11596962; doi:10.3390/microorganisms12112274)
Supplement: Supplementary file 1 [file microorganisms-12-02274-s001.zip › Supplementary Figure S1-S3.pdf]

## Supplementary figure captions

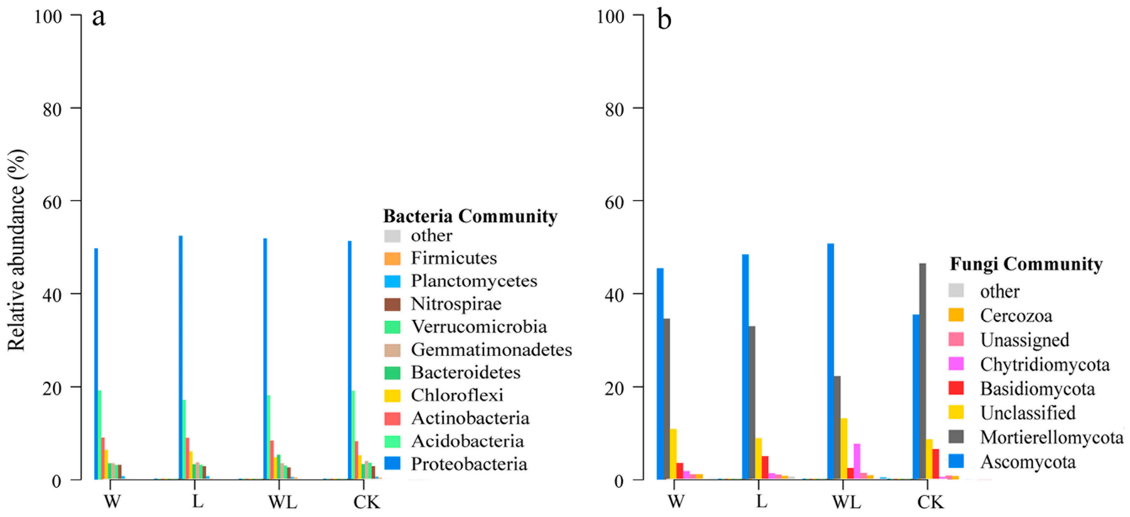


Figure S1. Changes in relative abundance of microbial communities under different treatments. Bacteria community (a). Fungi community (b). WL, Warming + Litter removal; W, Warming; L, Litter removal; CK, Control.

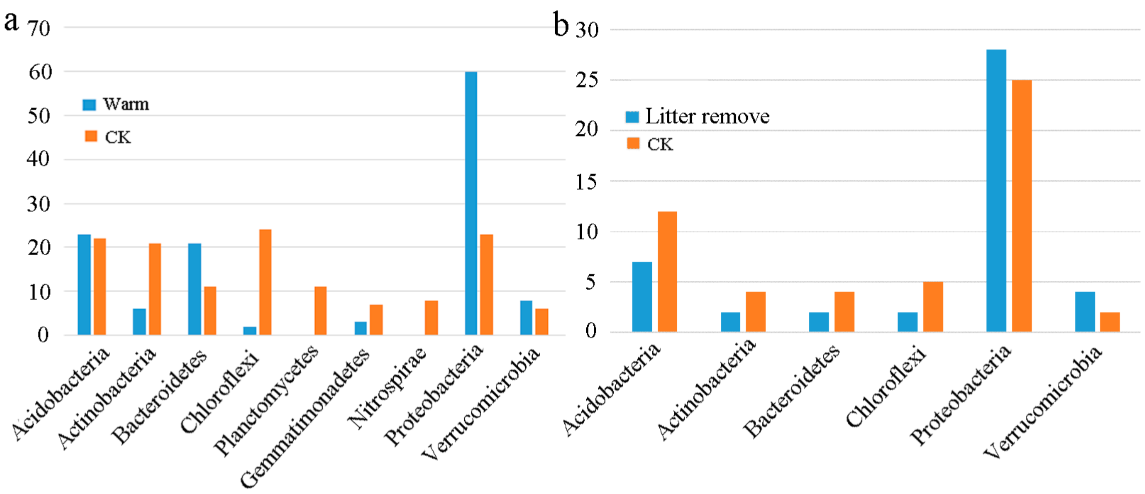


Figure S2. Specific microbial communities in soil under different treatments. Number of specific OTUs in warming(W,WL) and control(CK) treatments (a). Number of specific OTUs in litter removal(L,WL) and control(CK) treatments (b).

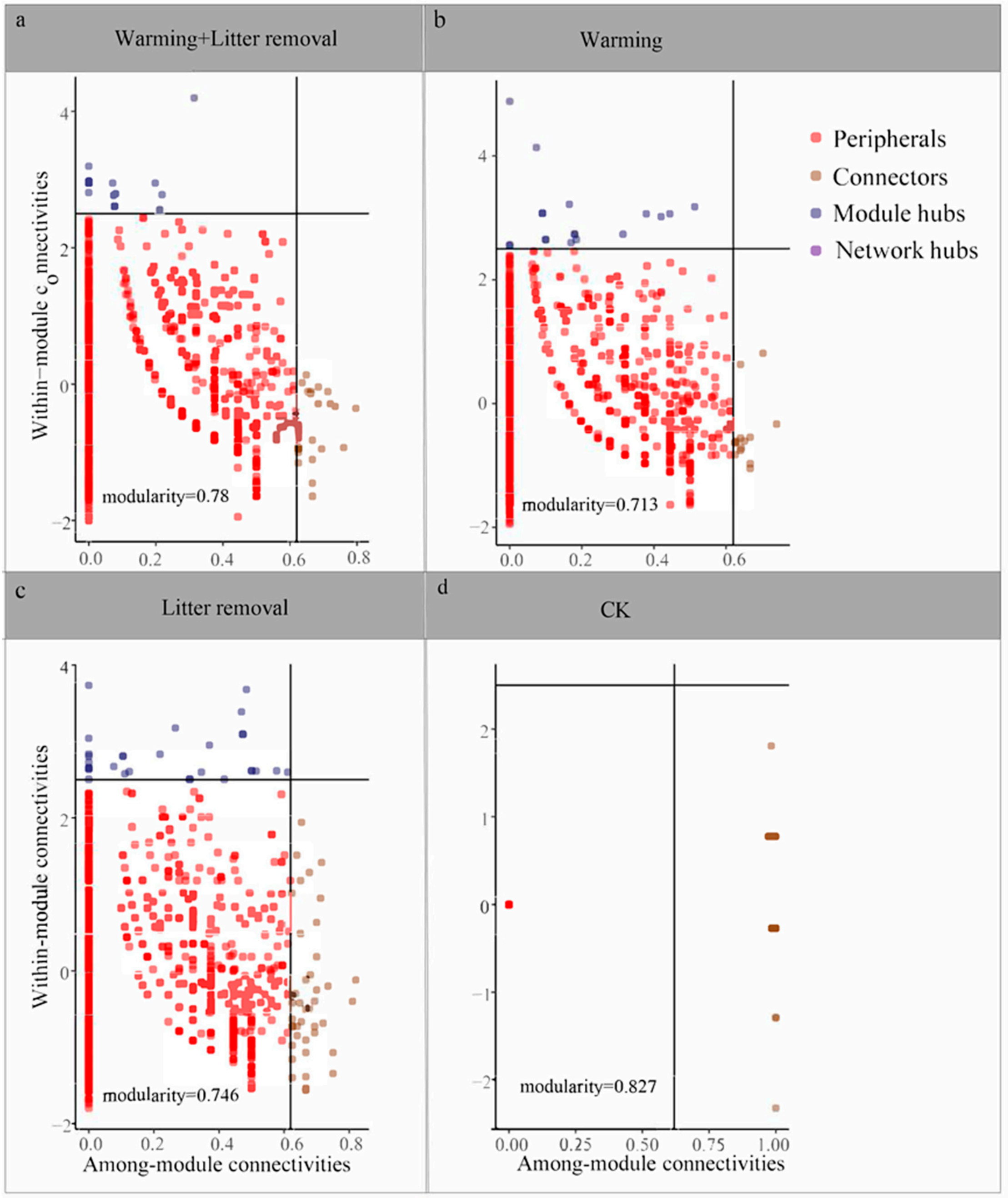


Figure S3 Zi-Pi plots show the distributions of OTUs based on their topological roles in fungi and bacteria networks. According to zi = 2.5 and pi = 0.62, all nodes are divided into 4 categories: peripherals, connectors, module hubs, and network hubs. WL, Warming + Litter removal; W, Warming; L, Litter removal; CK, Control.
